# Association of Physical Activity and Socioeconomic Status With Glycaemic Control in Adults With Type 1 Diabetes: A Cross‐Sectional Study Using CGM Data

**DOI:** 10.1002/dmrr.70146

**Published:** 2026-02-27

**Authors:** Fernando Sebastian‐Valles, Rafael Simó, Jose Alfonso Arranz Martín, Mercè Abad, Alicia Justel Enriquez, Cristina Pérez‐Fernández, Sara Jiménez Blanco, Cristina Hernández, Mónica Marazuela, Olga Simó‐Servat

**Affiliations:** ^1^ Department of Endocrinology and Nutrition Hospital Universitario de La Princesa Instituto de Investigación Sanitaria de La Princesa Universidad Autónoma de Madrid Madrid Spain; ^2^ Endocrinology and Nutrition Department Vall d'Hebron University Hospital and Vall d'Hebron Research Institute Barcelona Spain; ^3^ Centro de Investigación Biomédica en Red de Diabetes y Enfermedades Metabólicas Asociadas (CIBERDEM) Instituto de Salud Carlos III (ICSIII) Madrid Spain

**Keywords:** IPAQ, physical activity, SES, time in range, type 1 diabetes

## Abstract

**Aims:**

This study examined the independent and combined effects of physical activity and socioeconomic status (SES) on glycaemic control in adults with T1D using continuous glucose monitoring (CGM).

**Methods:**

A cross‐sectional study included 423 adults with T1D from a public healthcare setting in whom physical activity was self‐assessed via the short‐form International Physical Activity Questionnaire (IPAQ). SES was estimated using mean annual net income by census tract. Glycaemic outcomes included time in range (TIR) and time in tight range (TITR), derived from CGM, and HbA1c. Multivariable linear models and four‐way mediation analyses were conducted.

**Results:**

Higher physical activity and income were independently associated with better glycaemic control. The highest activity quartile was associated with +8.0% TIR and −0.47% HbA1c (*p* < 0.01). Physical activity partially mediated the effect of income on TIR (pure indirect effect *β* = 2.42, *p* = 0.013), accounting for 23% of the total effect. No significant SES–activity interaction was observed. Physically active individuals also had a 16% lower insulin requirement and better lipid profile, independent of income. A modest TBR increase occurred without longer hypoglycaemia.

**Conclusion:**

Physical activity is associated with improved glycaemic and lipid control in T1D patients, regardless of income. Promoting physical activity might reduce SES‐related glycaemic disparities and improve outcomes.

## Graphical abstract

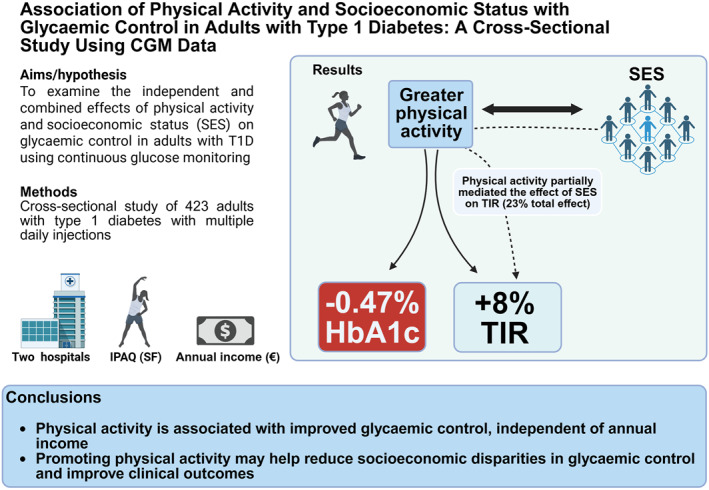

## 1 Introduction

Physical activity is a cornerstone of cardiometabolic health and a key component of type 1 diabetes (T1D) management [1]. The American Diabetes Association (ADA) recommends that adults with T1D engage in at least 150 min per week of moderate‐to‐vigorous aerobic exercise, distributed over a minimum of 3 days, with no more than two consecutive days without activity [2]. However, many adults with T1D do not meet these recommendations [3] and are often as inactive as the general population [4].

In individuals with T1D, regular physical activity has been associated with improved glycaemic control, reduced insulin requirements, favourable lipid profiles, and a decreased risk of both cardiovascular and microvascular complications [5, 6]. Despite these benefits, participation in physical activity remains suboptimal, often limited by fear of hypoglycaemia, low confidence in adjusting insulin or carbohydrate intake around exercise, and structural barriers such as inadequate support from healthcare professionals and lack of access to safe environments [3, 7].

Socioeconomic status (SES) is a well‐established determinant of health outcomes in T1D, influencing glycaemic control [8, 9], the risk of diabetes‐related complications [10, 11], and even mortality [12]. Individuals with lower SES are more likely to face barriers to effective self‐management, including limited access to healthy foods, health education, and opportunities for physical activity [13]. Although low SES has been linked to lower physical activity levels in people with T1D [14, 15], it remains unclear whether the association between physical activity and CGM‐derived glycaemic outcomes differs across socioeconomic strata, and to what extent SES‐related differences in glycaemic control may be partly explained by disparities in physical activity. To the best of our knowledge, no studies have addressed this question using measures of glucose control such as CGM. Importantly, this scientific gap is also relevant in universal public healthcare systems, where access to glucose monitoring technology may be more equitable.

The objective of this study was to examine the association between physical activity and SES, and to evaluate their independent and combined effects on glycaemic control in adults with T1D treated with multiple daily insulin injections and using intermittently scanned continuous glucose monitoring (isCGM) within a universal public healthcare system.

## 2 Methods

### 2.1 Study Design and Participants

We conducted a cross‐sectional observational study in a cohort of adults with T1D receiving outpatient care at two university‐affiliated public hospitals (Hospital Universitario de La Princesa (Madrid, Spain) and Hospital Universitario Vall d'Hebron (Barcelona, Spain) between October 2024 and May 2025. Of the 425 individuals initially screened, 423 consecutive participants were included based on the availability of complete data from the International Physical Activity Questionnaire (IPAQ, short form) [16].

Inclusion criteria were: confirmed diagnosis of T1D, age ≥ 18 years, and use of continuous glucose monitoring system FreeStyle Libre 2 (Abbott) with at least 70% valid glucose data during the two weeks preceding the clinical visit [17]. This device was the first intermittently scanned CGM system to be publicly funded in Spain in 2019 and is therefore the most widely used in clinical practice and observational studies in this setting [18, 19]. Patients using insulin pumps or other CGM systems were excluded to ensure a homogeneous study population. The study was approved by the Research Ethics Committee of Hospital Universitario de La Princesa (study ID: 5714‐18/24) and conducted in accordance with the principles of the Declaration of Helsinki. All participants provided written informed consent. The study adheres to the Strengthening the Reporting of Observational Studies in Epidemiology (STROBE [20]) and A Guideline for Reporting Mediation Analyses of Randomized Trials and Observational Studies: The AGReMA Statement [21] (Tables S1 and S2).

### 2.2 Exposures: Socioeconomic Status and Physical Activity

SES was assessed using the mean annual net income per capita of each participant's residential census tract, as reported in the 2022 Household Income Distribution Atlas published by the Spanish National Statistics Institute. This variable shows a strong correlation with the 2011 Spanish deprivation index [22, 23, 24] and was used as a contemporary proxy for SES.

Physical activity was measured using the International Physical Activity Questionnaire (IPAQ, short form [16]. Weekly metabolic equivalents (METs/week) were calculated as a continuous variable and also stratified into quartiles for comparative analyses.

### 2.3 Sample Size

In the study population, a mean TIR of approximately 60% was expected [22]. Based on international guidelines [17], the minimal clinically important difference for comparing mean TIR values is defined as 5%. Therefore, assuming a two‐sided α error of 5% and a statistical power of 80%, the estimated sample size required for the study was approximately 396 participants.

### 2.4 Outcomes and Data Collection

Glucose metrics were extracted from the Libreview platform based on data generated by the FreeStyle Libre 2. The primary glycaemic outcomes were time in range (TIR, 70–180 mg/dL), time in tight range (TITR, 70–140 mg/dL), and HbA1c. The following glycaemic outcomes were collected: time in range (TIR, 70–180 mg/dL), time in tight range (TITR, 70–140 mg/dL), time below range (TBR, < 70 mg/dL), time above range (TAR, > 180 and >250 mg/dL), sensor usage, coefficient of variation (CV), standard deviation (SD), number of hypoglycaemic episodes, and duration of hypoglycaemia. Additional sociodemographic, clinical, and laboratory data were collected: age, sex, duration of diabetes, body mass index (BMI), smoking status, daily insulin dose per kilogram, most recent HbA1c (within 3 months), and lipid profile (total cholesterol, LDL, HDL, triglycerides, and remnant cholesterol). Diabetes‐related complications, including retinopathy, nephropathy, polyneuropathy, ischaemic heart disease, ischaemic stroke, and diabetic foot, were also recorded. HbA1c was measured using ion‐exchange high‐performance liquid chromatography (ADAMS A1c HA8180 V, ARKRAY). Total cholesterol was determined enzymatically (Alinity C Cholesterol Reagent Kit, Abbott). Remnant cholesterol was estimated by subtracting LDL and HDL cholesterol from total cholesterol [25]. Secondary outcomes were hypoglycaemia metrics (TBR, number and duration of episodes), daily insulin dose per kilogram, and lipid parameters.

### 2.5 Statistical Analysis

Continuous variables were described using medians and interquartile ranges (IQR), while categorical variables were reported as absolute and relative frequencies. Group comparisons were performed using the Kruskal–Wallis test for continuous variables and the chi‐squared test for categorical variables. Normality was assessed using the Kolmogorov–Smirnov test and normal probability plots. Spearman correlation analyses were used to explore bivariate associations between physical activity, SES, and glycaemic metrics (TIR, TITR, and HbA1c). For multivariable analyses, linear regression models were constructed adjusting for age, sex, diabetes duration, BMI, smoking status, insulin dose per kilogram, and LDL cholesterol levels. Primary multivariable models evaluated the independent associations of SES (annual income) and physical activity (METs) with TIR, TITR, and HbA1c. Secondary multivariable models evaluated associations with hypoglycaemia metrics, insulin dose per kilogram, and lipid parameters. Variables with skewed distributions were log‐transformed prior to modelling. Given the low rate of missing data ‐most of which occurred in outcome variables‐no imputation procedures were performed.

### 2.6 Interaction and Mediation Analyses

To evaluate whether the effect of physical activity on glycaemic control differed by SES, linear regression models including interaction terms between income and METs were constructed. Additionally, mediation analysis was performed using the four‐way decomposition method proposed by VanderWeele [26], which disaggregates the total effect into:

1. Controlled Direct Effect: the effect of income on TIR while holding physical activity constant.
2. Reference Interaction: the portion of the effect attributable to the interaction between income and physical activity, assuming no causal path from income to METs.
3. Mediated Interaction: the joint effect where income influences physical activity levels, which in turn modify the impact of income on TIR.
4. Pure Indirect Effect: the effect of income on TIR mediated solely through physical activity, without residual direct effects.

Two distinct sensitivity analyses were performed. First, the four‐way decomposition models were re‐estimated with additional adjustment for BMI and smoking status, beyond the covariates previously specified, to assess the robustness of the mediation–interaction effects. Second, estimates of direct, indirect, reference, and mediated interaction effects were independently evaluated using the classical Baron and Kenny approach, with statistical significance assessed by the Sobel test [27]. Mediated proportions were expressed using the RIT (relative to the total effect) and RID (relative to the direct effect) metrics. All analyses were conducted using Stata v17.0, and a two‐tailed *p*‐value < 0.05 was considered statistically significant.

### 2.7 Use of AI

Language editing assistance (academic English) was supported by an AI tool. No AI tools were used for data generation, statistical analysis or interpretation of results.

## 3 Results

### 3.1 Sample Characteristics

**FIGURE 1:**
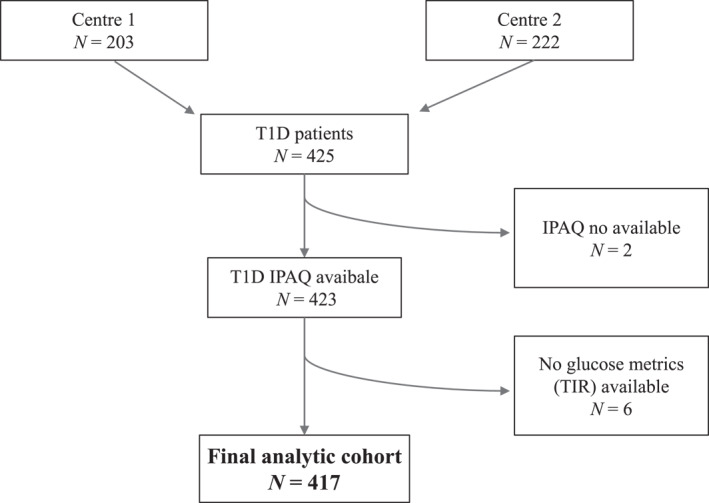
Flowchart of patient selection and inclusion in the study cohort. IPAQ, International Physical Activity Questionnaire; T1D, Type 1 Diabetes; TIR, Time in range (70–‐180 mg/dL).

From an initial cohort of 425 participants, complete IPAQ (short form) data were obtained from 423 individuals. The median age was 48.1 years (IQR: 35.7–59.4), with 44.9% being women. The median diabetes duration was 20.3 years (IQR: 9.6–32.5), and the median HbA1c was 7.4% (57 mmol/mol) [IQR: 6.8% (51 mmol/mol)–8.1% (65 mmol/mol)]). TIR (70–180 mg/dL) was available for 417 participants, with a median value of 62% (IQR: 49–75), and TITR; (70–140 mg/dL) had a median value of 38% (IQR: 26–50). The participant selection flowchart is shown in Figure 1, and baseline characteristics are summarised in Table 1.

**TABLE 1:** Clinical and glycaemic characteristics according to quartiles of physical activity.

Variable	*n*	Overall sample	METs Q1 < 693 METs/week	METs Q2 693–1520 METs/week	METs Q3 1521–2978 METs/week	METs Q4 > 2978 METs/week	*p* value
Age (years)	423	48.1 (35.7–59.4)	46.2 (36.2–57.4)	49.1 (35.1–59.1)	48.8 (35.9–59.6)	47.6 (35.9–62.6)	0.823
Sex (female)	423	190 (44.9%)	55 (51.4%)	54 (51.4%)	37 (34.9%)	44 (41.9%)	0.040
Duration of diabetes (years)	423	20.3 (9.6–32.5)	22.4 (11.9–36.9)	21.6 (9.9–31.4)	20.1 (9.3–28.6)	16.4 (7.6–32.6)	0.098
HbA1c ((%)[mmol/mol])	421	7.4 (6.8–8.1) [57 (51–65)]	7.7 (7.1–8.5) [61 (54–69)]	7.7 (6.9–8.2) [61 (52–66)]	7.4 (6.8–8.1) [57 (51–65)]	6.9 (6.5–7.5) [52 (48–58)]	< 0.001
Smoking habit	419	135 (32.2%)	40 (37.4%)	45 (43.7%)	29 (27.4%)	21 (20.4%)	0.002
Lipid–lowering treatment	423	192 (45.4%)	52 (48.6%)	46 (43.8%)	51 (48.1%)	43 (41.0%)	0.637
BMI kg/m^2^	418	25.1 (22.6–28.3)	26.4 (23.3–30.3)	24.9 (22.6–28.3)	24.6 (22.1–27.3)	25.1 (22.6–27.5)	0.823
Insulin IU/kg/day	421	0.55 (0.43–0.67)	0.56 (0.45–0.69)	0.54 (0.45–0.71)	0.56 (0.42–0.69)	0.51 (0.40–0.62)	0.040
Annual income (€)	423	16,099 (12,497–21,852)	13,403 (11,757–17,483)	15,941 (12,386–20,604)	16,888 (12,771–21,852)	19,696 (14,968–24,125)	< 0.001
Average glucose	416	160 (142–182)	170 (152–198)	161 (145–183)	158 (141–183)	148 (134–165)	< 0.001
CV	413	35 (31–40)	36 (31–39)	36 (31–42)	35 (30–39)	35 (30–40)	0.684
SD	413	57.4 (45.3–68.1)	60.3 (46.6–72.5)	59.4 (46.5–71.5)	56.3 (45.2–65.7)	52.6 (43.5–63.2)	0.016
GMI	410	7.1 (6.7–7.7)	7.4 6.9–8	7.2 (6.8–7.7)	7.1 (6.7–7.7)	6.9 (6.5–7.3)	< 0.001
TIR (70–180 mg/dL)	417	62 (49–75)	58 (42–68)	61 (46–73)	61 (48–75)	68 (58–81)	< 0.001
TITR (70–140 mg/dL)	398	38 (26–50)	32 (21–43)	38 (26–48)	37 (27–50)	44 (33–56)	< 0.001
TAR > 180 mg/dL	412	26 (16–34)	27 (22–34)	27 (18–35)	25 (6–37)	22 (12–30)	0.001
TAR > 250 mg/dL	418	8 (3–18)	11 (5–25)	8 (4–19)	8 (3–18)	5.5 (1.5–13)	0.001
TBR < 70 mg/dL	415	2 (1–6)	2 (1–4)	2 (1–6)	2 (1–5)	3 (1–6)	0.192
Sensor use (%)	418	97 (93–98)	97 (93–98)	96 (92–98)	97 (94–99)	97 (94–98)	0.070
Number of hypoglycaemic episodes	394	6 (2–11)	6 (1–9)	6 (2–12)	5 (2–12)	8 (3–13)	0.202
Duration of hypoglycaemic episodes (minutes)	393	80 (50–117)	80 (55–116)	91 (53–116)	73 (45–109)	81 (52–126)	0.283
Total cholesterol (mg/dL)	415	172 (149–199)	176 (53–202)	175 (152–202)	169 (155–193)	165 (141–199)	0.426
LDL‐cholesterol (mg/dL)	389	96 (76–118)	102 (78–121)	96 (78–122)	92 (77–113)	94 (68–116)	0.284
HDL‐cholesterol (mg/dL)	388	56 (47–67)	52 (44–64)	54 (47–65)	59 (51–67)	57 (48–70)	0.013
Triglycerides (mg/dL)	410	72 (54–96)	84 (69–118)	70 (56–103)	65 (52–85)	66 (46–90)	< 0.001
Remnant‐cholesterol (mg/dL)	388	14 (11–19)	17 (14–24)	14 (11–19)	13 (10–17)	13 (9–18)	< 0.001

Abbreviations: BMI, body mass index; CV, coefficient of variation; GMI, glucose management indicator; HbA1c, glycated haemoglobin; METs, metabolic equivalents of task; SD, standard deviation; TAR, time above range; TBR, time below range; TIR, time in range (70–180 mg/dL); TITR, time in tight range (70–140 mg/dL).

### 3.2 Primary Objective: Association of SES and Physical Activity With Glycaemic Outcomes

#### 3.2.1 Bivariate Associations

**FIGURE 2:**
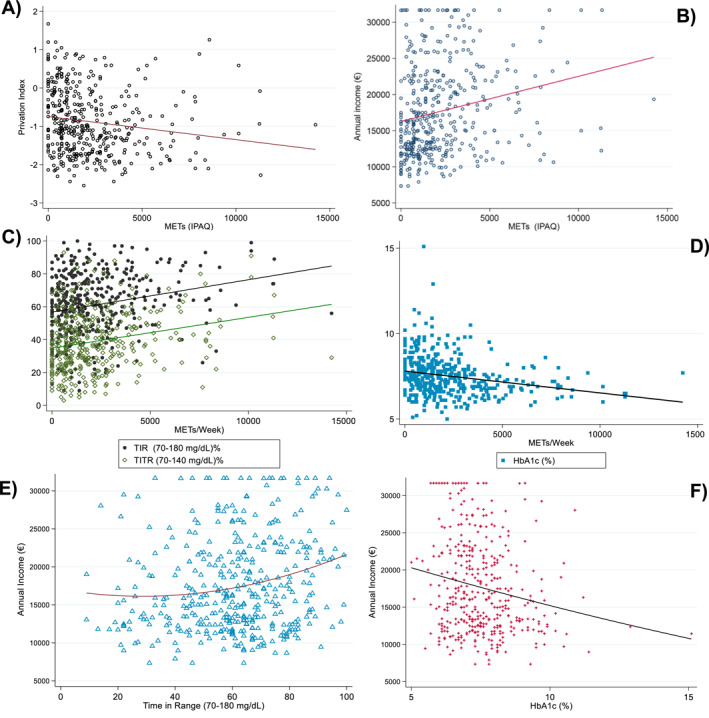
Correlations between socioeconomic status and physical activity (a, b), glycaemic metrics and physical activity (c, d) and socioeconomic status and glycaemic metrics (e and f). Socioeconomic status (a and b), and glycaemic metrics. Scatter plots illustrating the associations between physical activity (measured in METs/week) socioeconomic status (proxied by annual income and the deprivation index), and glycaemic control metrics in adults with type 1 diabetes. (A) Inverse correlation between METs/week and deprivation index (Spearman's *ρ* = −0.235; *p* < 0.001). (B) Positive correlation between METs/week and annual income (*ρ* = 0.271; *p* < 0.001). (C) Positive correlations between METs/week and time in range (TIR, 70–180 mg/dL; *ρ* = 0.237; *p* < 0.001) and time in tight range (TITR, 70–140 mg/dL; *ρ* = 0.239; *p* < 0.001). (D) Inverse correlation between METs/week and HbA1c (*ρ* = −0.276; *p* < 0.001). (E) Positive association between annual income and TIR (*ρ* = 0.168; *p* < 0.001). (F) Inverse association between annual income and HbA1c (*ρ* = −0.173; *p* < 0.001). All associations were evaluated using Spearman rank correlation coefficients. Trends suggest that both higher physical activity and higher income are associated with more favourable glycaemic outcomes. HbA1c, Glycated haemoglobin; METs, metabolic equivalents of task; TIR, time in range (70–180 mg/dL); TITR, time in tight range (70–140 mg/dL).

Spearman correlation analyses were performed to examine the relationships between physical activity, SES, and continuous glucose control metrics. Annual income was positively correlated with physical activity levels (Spearman's rho = 0.271; *p* < 0.001) and inversely correlated with the deprivation index (rho = −0.235; *p* < 0.001). METs/week were positively correlated with TIR (rho = 0.237; *p* < 0.001) and TITR (rho = 0.239; *p* < 0.001), and inversely with HbA1c (rho = −0.276; *p* < 0.001). Similarly, net income was positively associated with TIR (rho = 0.168; *p* < 0.001) and inversely with HbA1c (rho = −0.173; *p* < 0.001). These associations are illustrated in Figure 2.

#### 3.2.2 Participant Characteristics by Physical Activity Quartiles

When stratified by weekly MET quartiles, higher physical activity levels were associated with a lower proportion of women and a reduced prevalence of smoking. Participants in the highest quartile showed higher TIR and TITR, lower HbA1c, lower TAR > 180 and >250 mg/dL, lower glycaemic standard deviation, and reduced insulin/kg per day. This group also had a more favourable lipid profile, with lower triglycerides and remnant cholesterol, and higher HDL cholesterol, without significant differences in lipid‐lowering therapy use across quartiles. These results are presented in Table 1. There was a non‐significant trend towards a lower prevalence of diabetic polyneuropathy in the most active quartile (Figure S1)

#### 3.2.3 Multivariable Associations

**FIGURE 3:**
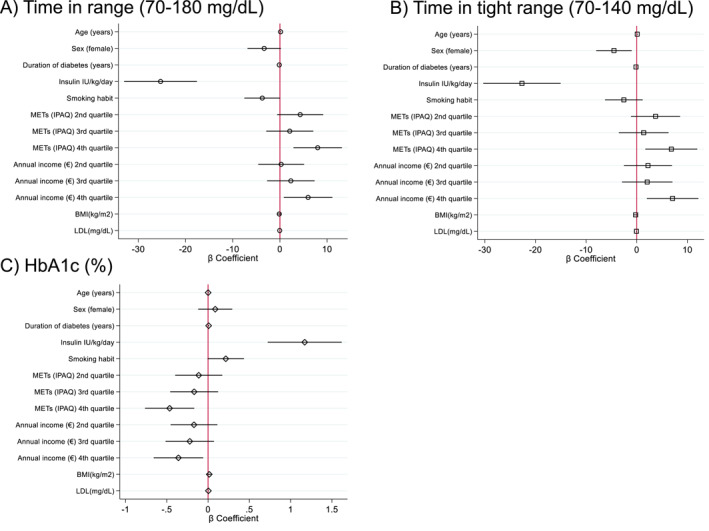
Multivariable associations between physical activity, socioeconomic status, and glycaemic outcomes. Forest plots display β coefficients and 95% confidence intervals from multivariable linear regression models assessing the independent associations of physical activity (measured as metabolic equivalents [METs] using the IPAQ) and annual income (as a proxy of socioeconomic status) with (A) TITR, (B) TIR, and (C) HbA1c levels in adults with type 1 diabetes. Models were adjusted for age, sex, diabetes duration, daily insulin dose (IU/kg/day), smoking habit, body mass index, and LDL cholesterol. Higher quartiles of METs and annual income were independently associated with increased TITR and TIR and lower HbA1c. These findings suggest that both physical activity and socioeconomic status may contribute to improved glycaemic control. HbA1c, Glycated haemoglobin; METs, metabolic equivalents of task; TIR, time in range (70–180 mg/dL); TITR, time in tight range (70–140 mg/dL).

Multivariable linear regression models were used to evaluate the associations of METs and annual income with TIR, TITR, and HbA1c. After adjustment for age, sex, diabetes duration, BMI, smoking, insulin dose per kg, and LDL cholesterol, both higher physical activity and higher SES (income quartiles) were independently associated with improved glycaemic control. Compared to the lowest quartile, the highest physical activity quartile was associated with increased TIR (+8.0% points; 95% CI: 2.9 to 13.1; *p* = 0.002), TITR (+6.8; 95% CI: 1.7 to 11.9; *p* = 0.009), and lower HbA1c (−0.47% [–5 mmol/mol]; 95% CI: −0.76% [–8 mmol/mol] to −0.17% [–2 mmol/mol]; *p* = 0.002). Similarly, individuals in the highest income quartile had significantly better TIR (+6.0 points; 95% CI: 0.8 to 11.1; *p* = 0.023), TITR (+7.1; 95% CI: 2.0 to 12.2; *p* = 0.006), and lower HbA1c (−0.36% [–4 mmol/mol]; 95% CI: −0.66% [–7 mmol/mol] to −0.06% [–1 mmol/mol]; *p* = 0.019 (Figure 3).

#### 3.2.4 Interaction and Mediation Analyses

**FIGURE 4:**
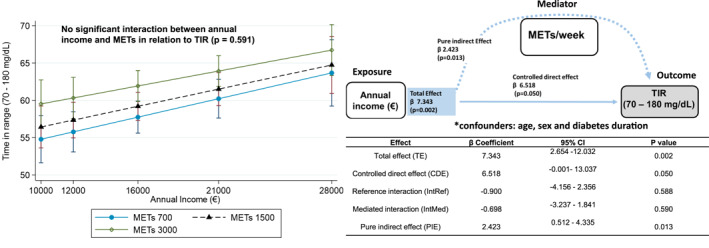
Physical activity does not modify the association between annual income and time in range, but partially mediates its effect. The figure illustrates the association between annual income and time in range (TIR, 70–180 mg/dL), stratified by physical activity levels expressed in METs/week (700, 1500, and 3000 METs). The left panel shows that higher annual income is associated with increased TIR across all levels of physical activity, with no significant interaction between income and METs in relation to TIR (*p* = 0.591). This suggests that the beneficial impact of physical activity on glycaemic control is consistent across income strata. The right panel presents a directed acyclic graph (DAG) depicting the mediation model. It shows a pure indirect effect of METs/week on the relationship between annual income and TIR (*β* = 2.423, *p* = 0.013), indicating that physical activity partially mediates the effect of income on glycaemic control. The controlled direct effect of income on TIR remains significant (*β* = 6.518, *p* = 0.050), and the total effect of income on TIR is *β* = 7.343 (*p* = 0.002). Both models were adjusted for sex, age, and diabetes duration.

Given the observed association between annual income, physical activity (METs by IPAQ), and TIR, we explored whether the effect of exercise on TIR varied by SES using a multivariable model with an interaction term adjusted for age, sex, and diabetes duration. Both income and METs remained independently associated with TIR, but no significant interaction was observed (*β* = −3.3e–08; *p* = 0.591), suggesting no effect modification. Predicted margins confirmed a progressive increase in TIR with higher METs and income, without statistical interaction (Figure 4).

A mediation analysis using VanderWeele's four‐way decomposition revealed a significant pure indirect effect of physical activity on the income–TIR association (*β* = 2.42; *p* = 0.013), with no evidence of interaction (Figure 4). Sensitivity analysis using the Baron and Kenny approach confirmed partial mediation (*p* = 0.002), explaining approximately 23% of the total effect of income on TIR (Figure S2). When sensitivity analyses were performed with additional adjustment for BMI and smoking status, the results remained consistent with the initial findings (*β* = 2.48; *p* = 0.012) (Figure S2). These findings suggest that physical activity is associated with a consistent glucose‐lowering effect across SES strata, and that part of the SES‐related differences in TIR are attributable to disparities in physical activity levels.

### 3.3 Secondary Outcomes

#### 3.3.1 Hypoglycaemia and Physical Activity

Although univariable analyses showed no statistically significant differences in hypoglycaemia metrics across physical activity quartiles, multivariable linear regression models were fitted using log‐transformed outcomes due to non‐normal distributions (Figure S3). In fully adjusted models, individuals in the highest quartile of physical activity exhibited a significantly higher natural logarithm of the number of hypoglycaemic episodes compared with those in the lowest quartile (*β* = 0.331; 95% CI: 0.041 to 0.621; *p* = 0.025), after adjustment for age, sex, diabetes duration, body mass index, and socioeconomic status. No significant differences were observed for the intermediate physical activity quartiles.

In contrast, physical activity was not associated with the log‐transformed duration of hypoglycaemia. With respect to TBR < 70 mg/dL, participants in the highest MET quartile showed a higher log‐transformed TBR (*β* = 0.296; 95% CI: 0.018 to 0.575; *p* = 0.037), whereas no significant association was found with total hypoglycaemia duration. In these models, older age, female sex, and longer diabetes duration were independently associated with hypoglycaemia duration.

#### 3.3.2 Insulin Dose and Physical Activity

As shown in Table 1, participants in the highest physical activity quartile had lower insulin requirements per kilogram. In multivariable models adjusted for age, sex, diabetes duration, and income, the highest MET quartile was associated with a significant reduction in insulin/kg (*β* = −0.090; 95% CI: −0.154 to −0.026; *p* = 0.006), representing a 16.4% decrease compared to the cohort's median dose. This trend was not significant in intermediate quartiles. Additionally, female sex was independently associated with lower insulin requirements (*β* = −0.047; 95% CI: −0.092 to −0.003; *p* = 0.036), with no significant associations for age, diabetes duration, or income (Figures S4 and S5).

#### 3.3.3 Lipid Parameters and Physical Activity

In multivariable models adjusted for age, sex, diabetes duration, lipid‐lowering therapy, and net income, higher physical activity levels (top MET quartiles) were significantly associated with improved lipid profiles. Compared to the lowest quartile, participants in quartile 4 had lower triglycerides (*β* = −19.34; *p* = 0.003), lower remnant cholesterol (*β* = −4.60; *p* = 0.002), and higher HDL cholesterol (*β* = 5.62; *p* = 0.015). These associations were also present in intermediate quartiles. No significant associations were found between physical activity and total cholesterol or LDL cholesterol. Full results are provided in Figure S6.

## 4 Discussion

The main finding of this study is that physical activity is associated with clinically meaningful improvements in CGM‐derived glycaemic control and cardiometabolic profile in adults with type 1 diabetes, and these benefits appear consistent across socioeconomic strata. Specifically, both physical activity and SES were independently associated with TIR, TITR, and HbA1c, and we found no evidence of an SES‐by‐physical activity interaction, indicating that the glycaemic benefits of physical activity were comparable across socioeconomic strata. Importantly, our mediation framework indicates that differences in physical activity levels may contribute to the SES gradient in TIR, thus suggesting that physical activity could be a feasible and cost‐effective target to attenuate SES‐related glycaemic disparities within a universal public healthcare context. Taken together, these findings support a clear equity‐oriented message: even in a setting with publicly funded CGM, socioeconomic differences in glycaemic outcomes persist and, therefore, behavioural intervention through physical activity promotion seems warranted.

These results align with prior studies documenting the metabolic benefits of exercise in people with T1D, including improved glycaemic outcomes and reduced cardiovascular risk [1, 28, 29]. However, our study builds upon existing evidence by using objective CGM‐based glucose metrics and applying a formal mediation framework, enabling quantification of the specific contribution of physical activity to SES‐related disparities. Unlike prior studies conducted in predominantly privately insured populations [14, 15], our data were collected within a universal public healthcare setting, which enhances its generalisability to systems where structural inequities might be mitigated through policy. Moreover, by incorporating TITR alongside TIR and HbA1c, we provide a granular description of glycaemic improvements that is directly interpretable for clinical practice and current CGM targets.

From a clinical perspective, the observed effects of physical activity on TIR (+8% points) and HbA1c (−0.47%, [–5 mmol/mol]) were both statistically and clinically significant, even after adjusting for SES and multiple covariates. Moreover, participants in the highest activity quartile had a 16% lower daily insulin requirement and more favourable lipid parameters, including lower triglycerides and remnant cholesterol. These secondary findings are directionally consistent with the expected physiological effects of exercise (improved insulin sensitivity and cardiometabolic risk profile) and reinforce the plausibility and coherence of the primary CGM‐based associations. This is particularly relevant given the growing evidence from Mendelian randomisation studies supporting a causal relationship between remnant cholesterol and cardiovascular disease [30, 31], and its association with microvascular complications such as diabetic nephropathy, retinopathy [32], and foot disease in T1D [33]. At the physiological level, exercise enhances insulin sensitivity and mitigates oxidative stress and mitochondrial dysfunction, both of which are frequent findings in individuals with poorly controlled T1D [34, 35, 36]. Notably, studies in physically active older adults with well‐controlled T1D have reported preserved or even better mitochondrial function compared to non‐diabetic controls [37]. Exercise has also been associated with prolonged partial remission (‘honeymoon period’) after T1D onset, suggesting a protective effect on β‐cell function [38].

A central contribution of this study is the integration of socioeconomic context with CGM outcomes to address a clinically actionable question: does physical activity confer similar glycaemic benefits across SES strata? The absence of a significant SES × physical activity interaction, together with the progressive increase in TIR across physical activity levels, suggests that the association between physical activity and glycaemic control is broadly consistent across socioeconomic strata. This is important because it supports the potential scalability of physical activity interventions without assuming differential effectiveness depending on SES. At the same time, the four‐way decomposition indicated a significant pure indirect effect, with approximately one‐quarter of the total association between SES and TIR explained by physical activity differences. This quantitative estimate provides a pragmatic framework for intervention design: reducing SES gaps in physical activity may translate into measurable improvements in glycaemic equity, even when technological access to CGM is universal.

As a minor caveat, our data showed a slight increase in TBR and number of hypoglycaemic episodes among individuals with the highest levels of physical activity; however, this was not accompanied by a longer duration of hypoglycaemic episodes. This finding could be explained by the widespread use of CGM alarms, which have been shown to mitigate exercise‐induced hypoglycaemia [39]. From a clinical standpoint, these results reinforce the need to pair physical activity counselling with structured education on insulin and carbohydrate adjustments around exercise, particularly for individuals with higher activity levels.

Despite all these benefits, adherence to physical activity remains suboptimal, often limited by fear of hypoglycaemia, lack of professional support, inadequate access to safe environments, and low confidence in insulin adjustments around exercise [3, 7]. Our findings imply that interventions aimed at promoting physical activity could improve glycaemic control across SES groups and serve as a powerful tool to reduce SES‐related health disparities. Moreover, the observed improvements in lipid profile—particularly reductions in triglycerides and remnant cholesterol, and increases in HDL—suggest a meaningful potential to lower cardiovascular risk. In parallel, the substantial reduction in insulin requirements among physically active individuals may translate into significant economic savings for both patients and healthcare systems, particularly in resource‐constrained settings. Given that many of the reported barriers are not unique to people with T1D [40] but are also common in the general population, scalable community‐based interventions‐such as supervised group programs, infrastructure enhancements, and digital support tools‐could be adapted and deployed effectively to promote physical activity in this vulnerable population. From an equity perspective, our findings support prioritising interventions that lower practical barriers to physical activity (tailored exercise education, accessible community programmes, and safe environments), which may complement technology‐access policies by addressing behavioural and environmental drivers of SES‐related differences in glycaemic outcomes.

This study has several limitations that should be acknowledged. Physical activity was assessed using a self‐reported questionnaire (IPAQ) rather than an accelerometer‐based measure, potentially overestimating activity levels [41]. As previously reported, IPAQ‐based estimates may overestimate true activity levels and are subject to recall and social desirability bias, which could have led to exposure misclassification and attenuation or inflation of associations. Notably, if measurement error in physical activity is largely non‐differential with respect to CGM outcomes, it would be expected to bias associations towards the null, suggesting that the observed estimates may be conservative. Second, we lacked information on certain potential confounders and contextual factors ‐such as alcohol intake and hypertension status‐ that may be associated with both physical activity and glycaemic outcomes. Third, the observed relationships between SES and physical activity may be affected by residual confounding from unmeasured individual‐ and neighbourhood‐level determinants (e.g., educational attainment, occupational characteristics, built environment, or health literacy), which could bias mediation estimates. In addition, SES was proxied using area‐level mean annual net income at the census‐tract level rather than individual‐level socioeconomic indicators. Although area‐based measures are widely used and correlate with deprivation indices, they may not fully capture within‐area heterogeneity and can introduce ecological misclassification. In principle, multilevel (hierarchical) modelling accounting for clustering by census tract could further disentangle individual‐ and area‐level effects; however, prior works [42, 43, 44, 45, 46] support the use of neighbourhood socioeconomic indicators as informative proxies for individual socioeconomic context in cardiometabolic research. We did not have complete, standardised CGM summaries over the preceding 12 months for all participants; therefore, we could not examine whether associations were consistent when using annual average CGM metrics, which would be valuable to reduce short‐term variability and better address temporality. Finally, the cross‐sectional design limits causal inference and temporality, particularly for mediation analyses, and reverse causation cannot be excluded (e.g., better glycaemic control enabling higher physical activity). Prospective longitudinal studies and intervention designs are therefore needed to confirm the directionality and magnitude of these associations. Accordingly, our mediation findings should be interpreted as quantifying associations under stated assumptions rather than as definitive causal effects.

In conclusion, physical activity is associated with better glycaemic and lipid control and lower insulin requirements in adults with T1D. Differences in physical activity account for nearly one‐fourth of the SES effect on glycaemic outcomes, and its benefits are consistent across income strata. Promoting physical activity and removing barriers to exercise might improve clinical outcomes, reduce healthcare costs, and help mitigate SES‐related disparities in T1D management. Future longitudinal and interventional studies should examine whether an increase in physical activity ‐particularly in lower‐SES contexts‐will result in sustained improvements in CGM targets and a reduction in SES‐related glycaemic outcomes.

## Author Contributions

**Fernando Sebastian‐Valles:** conceptualization, study design, supervision, formal analysis, writing – original draft. **Rafael Simó:** conceptualization, study design, writing – review and editing. **Jose Alfonso Arranz Martín:** data curation, investigation, writing – review and editing. **Mercè Abad:** software, data curation, writing – review and editing. **Alicia Justel Enríquez:** data curation, writing – review and editing. **Cristina Pérez‐Fernández:** software, data curation, writing – review and editing. **Sara Jiménez Blanco:** data curation, writing – review and editing. **Cristina Hernández:** supervision, writing – review and editing. **Mónica Marazuela:** supervision, writing – review and editing. **Olga Simó‐Servat:** conceptualization, study design, supervision, writing – original draft, writing – review and editing.

## Funding

This work was supported by PI19/00584 , PI22/01404 and PMP22/00,021 (funded by Instituto de Salud Carlos III) and co‐funded by FEDER funds to MM.

## Conflicts of Interest

The authors declare no conflicts of interest.

## Supporting information


Supporting Information S1

**Figure S1:** Distribution of chronic diabetes complications according to physical activity quartiles.

**Figure S2:** Four‐way decomposition and mediation analyses of the association between income and glycaemic control.

**Figure S3:** Hypoglycaemia metrics and association with physical activity.

**Figure S4:** Insulin requirements according to physical activity level.

**Figure S5:** Multivariable linear regression of insulin dose.

**Figure S6:** Association between physical activity and lipid parameters.

**Table S1:** STROBE Statement—checklist of items that should be included in reports of observational studies.

**Table S2:** AGReMA: A Guideline for Reporting Mediation Analyses.

## Data Availability

The data that support the findings of this study are available on request from the corresponding author. The data are not publicly available due to privacy or ethical restrictions.
