# Supplementary material for: Association of Physical Activity and Socioeconomic Status With Glycaemic Control in Adults With Type 1 Diabetes: A Cross‐Sectional Study Using CGM Data
Source: Diabetes Metab Res Rev. 2026 Feb 27;42(3):e70146. doi: 10.1002/dmrr.70146 (PMC12949369; doi:10.1002/dmrr.70146)
Supplement: Supplementary file 1 — Supporting Information S1 [file DMRR-42-e70146-s002.docx]

**Supplementary S1 Distribution of chronic diabetes complications according to physical activity quartiles**

**Supplementary S1** shows the distribution of chronic diabetes-related complications across quartiles of physical activity, expressed as metabolic equivalent minutes per week (METs/week) derived from the IPAQ questionnaire. Physical activity was categorized into quartiles as follows: Q1 (<693 METs/week), Q2 (693–1520 METs/week), Q3 (1521–2978 METs/week), and Q4 (>2978 METs/week). Data are presented as absolute frequencies and percentages. P values correspond to comparisons across METs quartiles.

**Supplementary S2. Four-way decomposition and mediation analyses of the association between income and glycaemic control**

**Supplementary S2** presents the four-way decomposition analysis assessing the association between net income and time in range (TIR), adjusted for age, sex, diabetes duration, body mass index, and smoking habit. The total effect was decomposed into the controlled direct effect, reference interaction, mediated interaction, and pure indirect effect mediated by physical activity.

Additionally, Panels A and B display mediation analyses conducted using the classical Baron and Kenny approach, including structural equation modeling results and estimates obtained using the medsem command, quantifying indirect, direct, and total effects, as well as the proportion mediated by physical activity.

**Supplementary S3. Hypoglycaemia metrics and association with physical activity**

**Supplementary S3** illustrates the non-normal distribution of hypoglycaemia-related variables, including time below range (TBR <70 mg/dL), number of hypoglycaemic episodes, and duration of hypoglycaemic episodes, justifying logarithmic transformation prior to regression analyses.

This supplement also reports multivariable analyses evaluating the association between physical activity quartiles (METs) and the natural logarithm of hypoglycaemia outcomes. Higher levels of physical activity were associated with a greater number of hypoglycaemic episodes and increased TBR, particularly in the highest METs quartile, without a corresponding increase in the duration of hypoglycaemic episodes. All models were adjusted for relevant sociodemographic and clinical covariates.

**Supplementary S4. Insulin requirements according to physical activity level**

**Supplementary S4** depicts the adjusted relationship between physical activity level, expressed as METs/week, and daily insulin requirements per kilogram of body weight. The curve represents predictions from a linear regression model including a quadratic term for physical activity, adjusted for age, sex, diabetes duration, and net income. Insulin requirements progressively decreased with increasing levels of physical activity. Vertical bars represent 95% confidence intervals for predicted insulin dose across MET levels ranging from 0 to 5000 METs/week. The overall model was statistically significant (p = 0.035).

**Supplementary S5. Multivariable linear regression of insulin dose**

**Supplementary S5** shows the results of a multivariable linear regression analysis evaluating the association between physical activity (METs quartiles), sociodemographic variables, and clinical factors with daily insulin dose expressed as units per kilogram of body weight (U/kg). Estimates are presented as β coefficients with 95% confidence intervals. The highest physical activity quartile was independently associated with lower insulin requirements.

**Supplementary S6. Association between physical activity and lipid parameters**

**Supplementary S6** presents multivariable linear regression analyses evaluating the adjusted association between physical activity quartiles (METs) and lipid parameters, including total cholesterol, LDL cholesterol, remnant cholesterol, HDL cholesterol, and triglycerides. Models were adjusted for age, sex, diabetes duration, lipid-lowering therapy, and annual income. Higher levels of physical activity were associated with lower remnant cholesterol and triglyceride concentrations, as well as higher HDL cholesterol levels.
