# Supplementary material for: Association of Physical Activity and Socioeconomic Status With Glycaemic Control in Adults With Type 1 Diabetes: A Cross‐Sectional Study Using CGM Data
Source: Diabetes Metab Res Rev. 2026 Feb 27;42(3):e70146. doi: 10.1002/dmrr.70146 (PMC12949369; doi:10.1002/dmrr.70146)
Supplement: Supplementary file 2 — Figure S1: Distribution of chronic diabetes complications according to physical activity quartiles. [file DMRR-42-e70146-s006.pptx]

## Slide 1
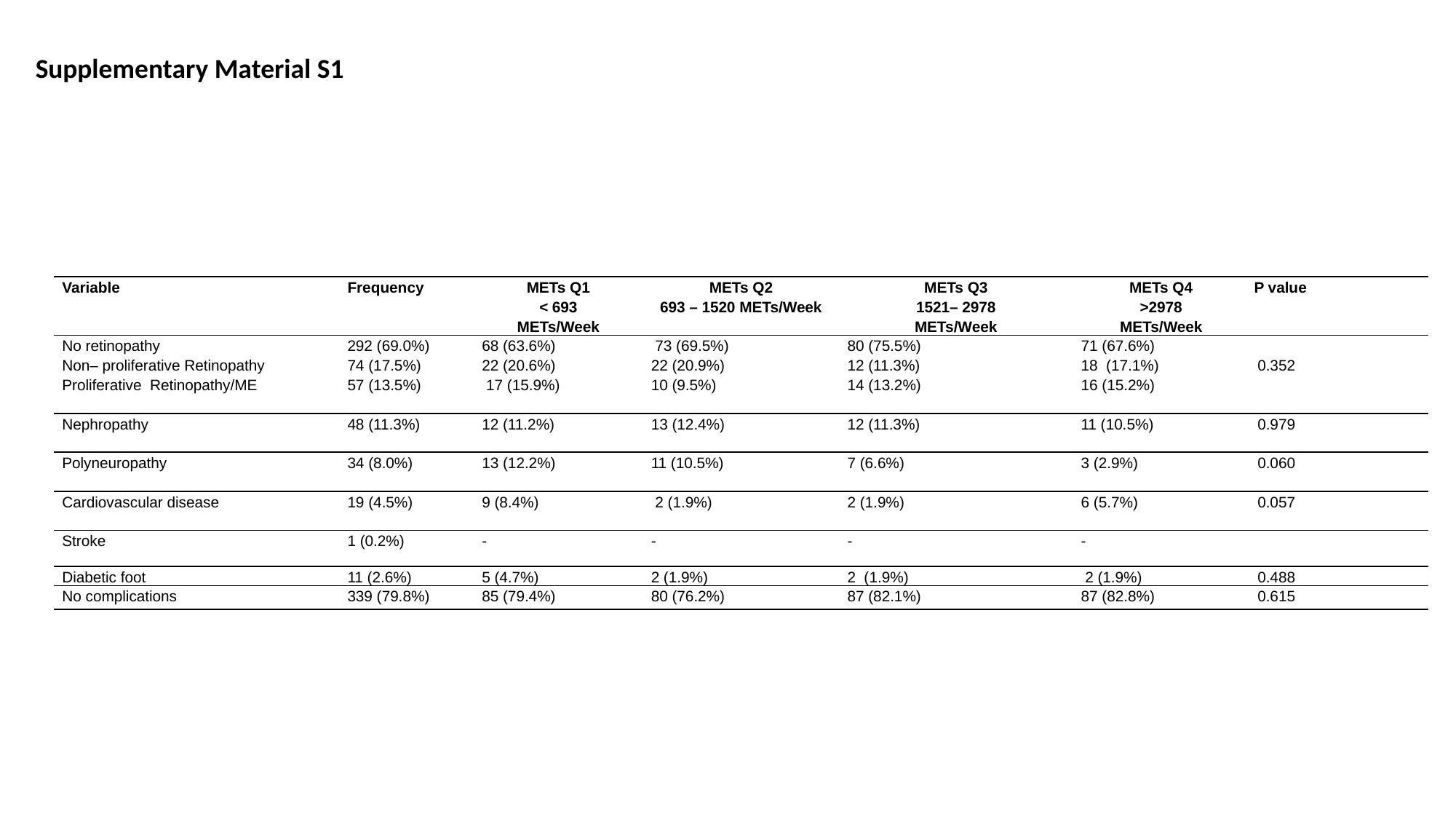

Supplementary Material S1
| Variable | Frequency | METs Q1 < 693 METs/Week | METs Q2 693 – 1520 METs/Week | METs Q3 1521– 2978 METs/Week | METs Q4 >2978 METs/Week | P value |
| --- | --- | --- | --- | --- | --- | --- |
| No retinopathy | 292 (69.0%) | 68 (63.6%) | 73 (69.5%) | 80 (75.5%) | 71 (67.6%) | |
| Non– proliferative Retinopathy | 74 (17.5%) | 22 (20.6%) | 22 (20.9%) | 12 (11.3%) | 18 (17.1%) | 0.352 |
| Proliferative Retinopathy/ME | 57 (13.5%) | 17 (15.9%) | 10 (9.5%) | 14 (13.2%) | 16 (15.2%) | |
| Nephropathy | 48 (11.3%) | 12 (11.2%) | 13 (12.4%) | 12 (11.3%) | 11 (10.5%) | 0.979 |
| Polyneuropathy | 34 (8.0%) | 13 (12.2%) | 11 (10.5%) | 7 (6.6%) | 3 (2.9%) | 0.060 |
| Cardiovascular disease | 19 (4.5%) | 9 (8.4%) | 2 (1.9%) | 2 (1.9%) | 6 (5.7%) | 0.057 |
| Stroke | 1 (0.2%) | - | - | - | - | |
| Diabetic foot | 11 (2.6%) | 5 (4.7%) | 2 (1.9%) | 2 (1.9%) | 2 (1.9%) | 0.488 |
| No complications | 339 (79.8%) | 85 (79.4%) | 80 (76.2%) | 87 (82.1%) | 87 (82.8%) | 0.615 |
