# Supplementary material for: Association of Physical Activity and Socioeconomic Status With Glycaemic Control in Adults With Type 1 Diabetes: A Cross‐Sectional Study Using CGM Data
Source: Diabetes Metab Res Rev. 2026 Feb 27;42(3):e70146. doi: 10.1002/dmrr.70146 (PMC12949369; doi:10.1002/dmrr.70146)
Supplement: Supplementary file 3 — Figure S2: Four‐way decomposition and mediation analyses of the association between income and glycaemic control. [file DMRR-42-e70146-s007.pptx]

## Slide 1
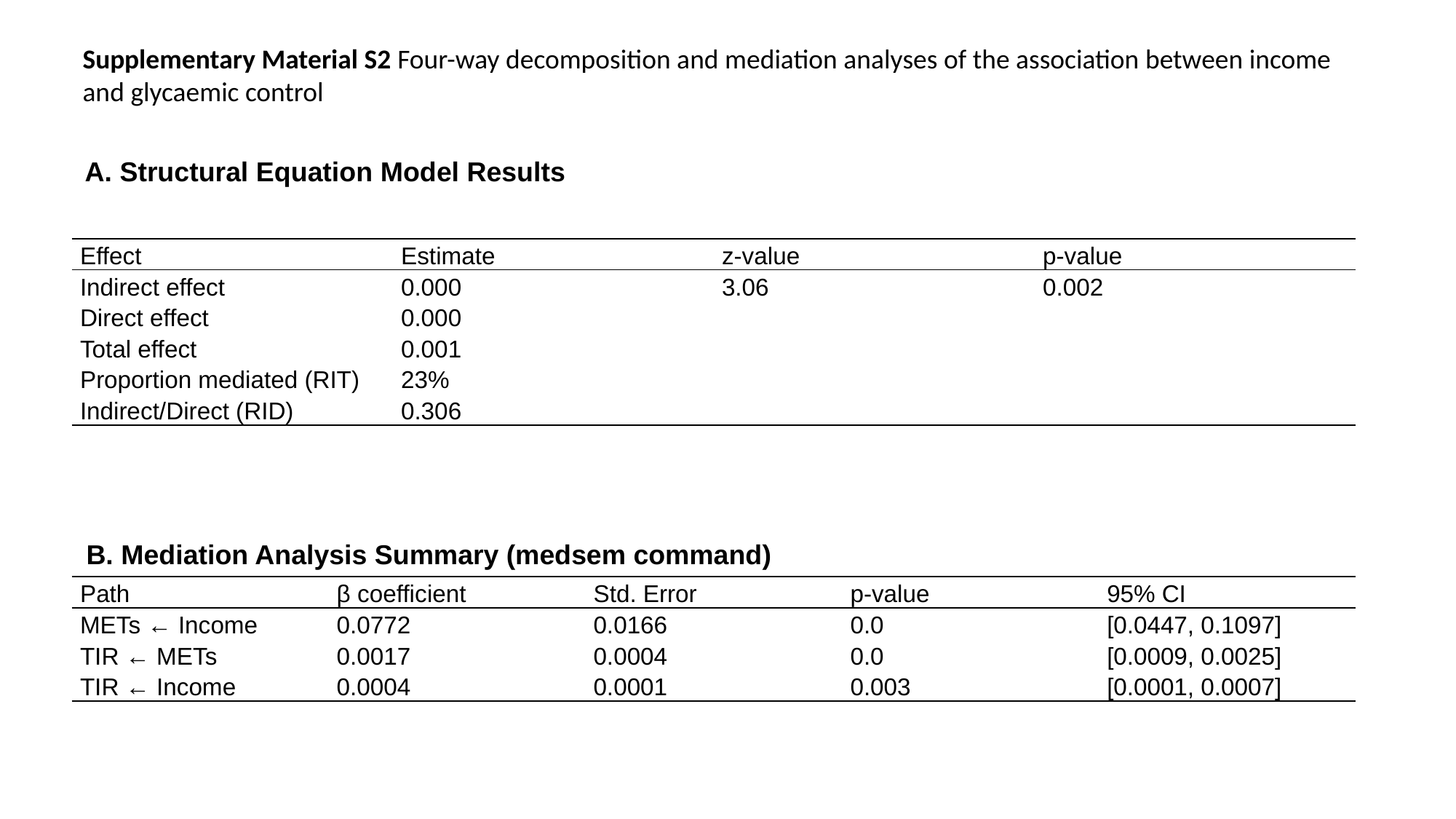

Supplementary Material S2 Four-way decomposition and mediation analyses of the association between income and glycaemic control
A. Structural Equation Model Results
| Effect | Estimate | z-value | p-value |
| --- | --- | --- | --- |
| Indirect effect | 0.000 | 3.06 | 0.002 |
| Direct effect | 0.000 | | |
| Total effect | 0.001 | | |
| Proportion mediated (RIT) | 23% | | |
| Indirect/Direct (RID) | 0.306 | | |
B. Mediation Analysis Summary (medsem command)
| Path | β coefficient | Std. Error | p-value | 95% CI |
| --- | --- | --- | --- | --- |
| METs ← Income | 0.0772 | 0.0166 | 0.0 | [0.0447, 0.1097] |
| TIR ← METs | 0.0017 | 0.0004 | 0.0 | [0.0009, 0.0025] |
| TIR ← Income | 0.0004 | 0.0001 | 0.003 | [0.0001, 0.0007] |
