# Supplementary material for: Association of Physical Activity and Socioeconomic Status With Glycaemic Control in Adults With Type 1 Diabetes: A Cross‐Sectional Study Using CGM Data
Source: Diabetes Metab Res Rev. 2026 Feb 27;42(3):e70146. doi: 10.1002/dmrr.70146 (PMC12949369; doi:10.1002/dmrr.70146)
Supplement: Supplementary file 4 — Figure S3: Hypoglycaemia metrics and association with physical activity. [file DMRR-42-e70146-s005.pptx]

## Slide 1
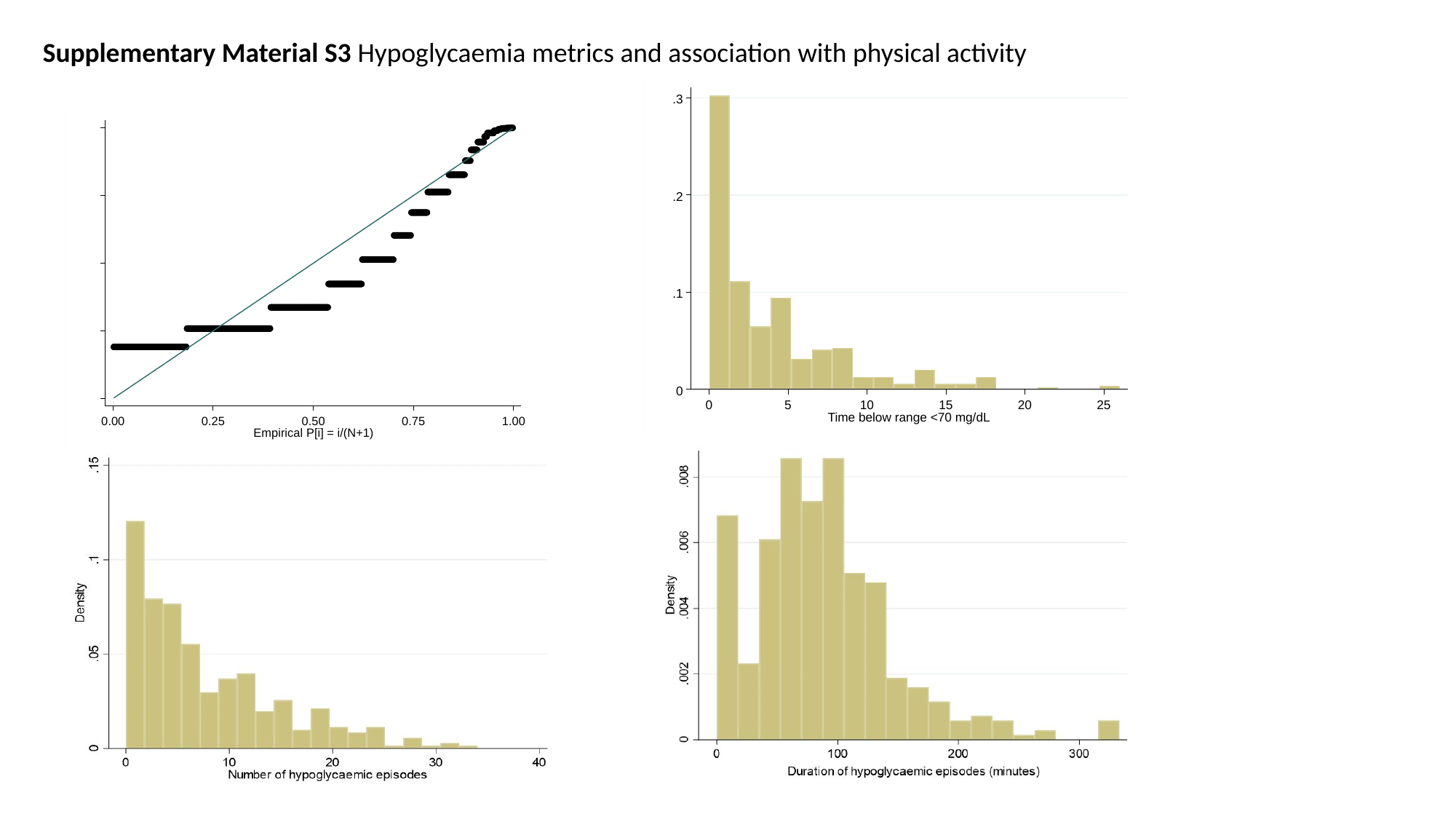

Supplementary Material S3 Hypoglycaemia metrics and association with physical activity

## Slide 2
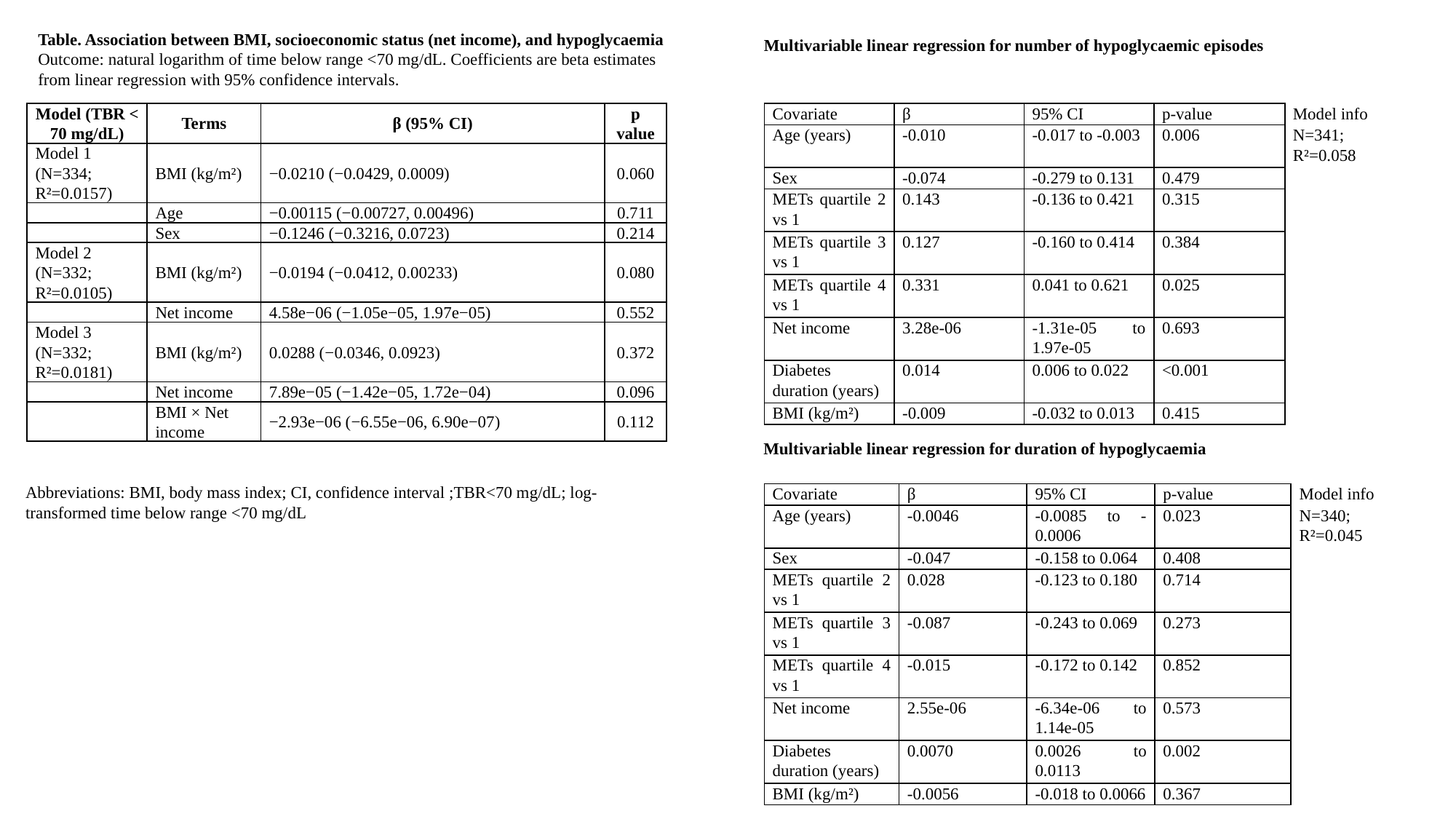

Table. Association between BMI, socioeconomic status (net income), and hypoglycaemia
Outcome: natural logarithm of time below range <70 mg/dL. Coefficients are beta estimates from linear regression with 95% confidence intervals.
Multivariable linear regression for number of hypoglycaemic episodes
| Model (TBR < 70 mg/dL) | Terms | β (95% CI) | p value |
| --- | --- | --- | --- |
| Model 1 (N=334; R²=0.0157) | BMI (kg/m²) | −0.0210 (−0.0429, 0.0009) | 0.060 |
| | Age | −0.00115 (−0.00727, 0.00496) | 0.711 |
| | Sex | −0.1246 (−0.3216, 0.0723) | 0.214 |
| Model 2 (N=332; R²=0.0105) | BMI (kg/m²) | −0.0194 (−0.0412, 0.00233) | 0.080 |
| | Net income | 4.58e−06 (−1.05e−05, 1.97e−05) | 0.552 |
| Model 3 (N=332; R²=0.0181) | BMI (kg/m²) | 0.0288 (−0.0346, 0.0923) | 0.372 |
| | Net income | 7.89e−05 (−1.42e−05, 1.72e−04) | 0.096 |
| | BMI × Net income | −2.93e−06 (−6.55e−06, 6.90e−07) | 0.112 |
| Covariate | β | 95% CI | p-value | Model info |
| --- | --- | --- | --- | --- |
| Age (years) | -0.010 | -0.017 to -0.003 | 0.006 | N=341; R²=0.058 |
| Sex | -0.074 | -0.279 to 0.131 | 0.479 | |
| METs quartile 2 vs 1 | 0.143 | -0.136 to 0.421 | 0.315 | |
| METs quartile 3 vs 1 | 0.127 | -0.160 to 0.414 | 0.384 | |
| METs quartile 4 vs 1 | 0.331 | 0.041 to 0.621 | 0.025 | |
| Net income | 3.28e-06 | -1.31e-05 to 1.97e-05 | 0.693 | |
| Diabetes duration (years) | 0.014 | 0.006 to 0.022 | <0.001 | |
| BMI (kg/m²) | -0.009 | -0.032 to 0.013 | 0.415 | |
Multivariable linear regression for duration of hypoglycaemia
Abbreviations: BMI, body mass index; CI, confidence interval ;TBR<70 mg/dL; log-transformed time below range <70 mg/dL
| Covariate | β | 95% CI | p-value | Model info |
| --- | --- | --- | --- | --- |
| Age (years) | -0.0046 | -0.0085 to -0.0006 | 0.023 | N=340; R²=0.045 |
| Sex | -0.047 | -0.158 to 0.064 | 0.408 | |
| METs quartile 2 vs 1 | 0.028 | -0.123 to 0.180 | 0.714 | |
| METs quartile 3 vs 1 | -0.087 | -0.243 to 0.069 | 0.273 | |
| METs quartile 4 vs 1 | -0.015 | -0.172 to 0.142 | 0.852 | |
| Net income | 2.55e-06 | -6.34e-06 to 1.14e-05 | 0.573 | |
| Diabetes duration (years) | 0.0070 | 0.0026 to 0.0113 | 0.002 | |
| BMI (kg/m²) | -0.0056 | -0.018 to 0.0066 | 0.367 | |
