# Supplementary material for: Association of Physical Activity and Socioeconomic Status With Glycaemic Control in Adults With Type 1 Diabetes: A Cross‐Sectional Study Using CGM Data
Source: Diabetes Metab Res Rev. 2026 Feb 27;42(3):e70146. doi: 10.1002/dmrr.70146 (PMC12949369; doi:10.1002/dmrr.70146)
Supplement: Supplementary file 5 — Figure S4: Insulin requirements according to physical activity level. [file DMRR-42-e70146-s003.pptx]

## Slide 1
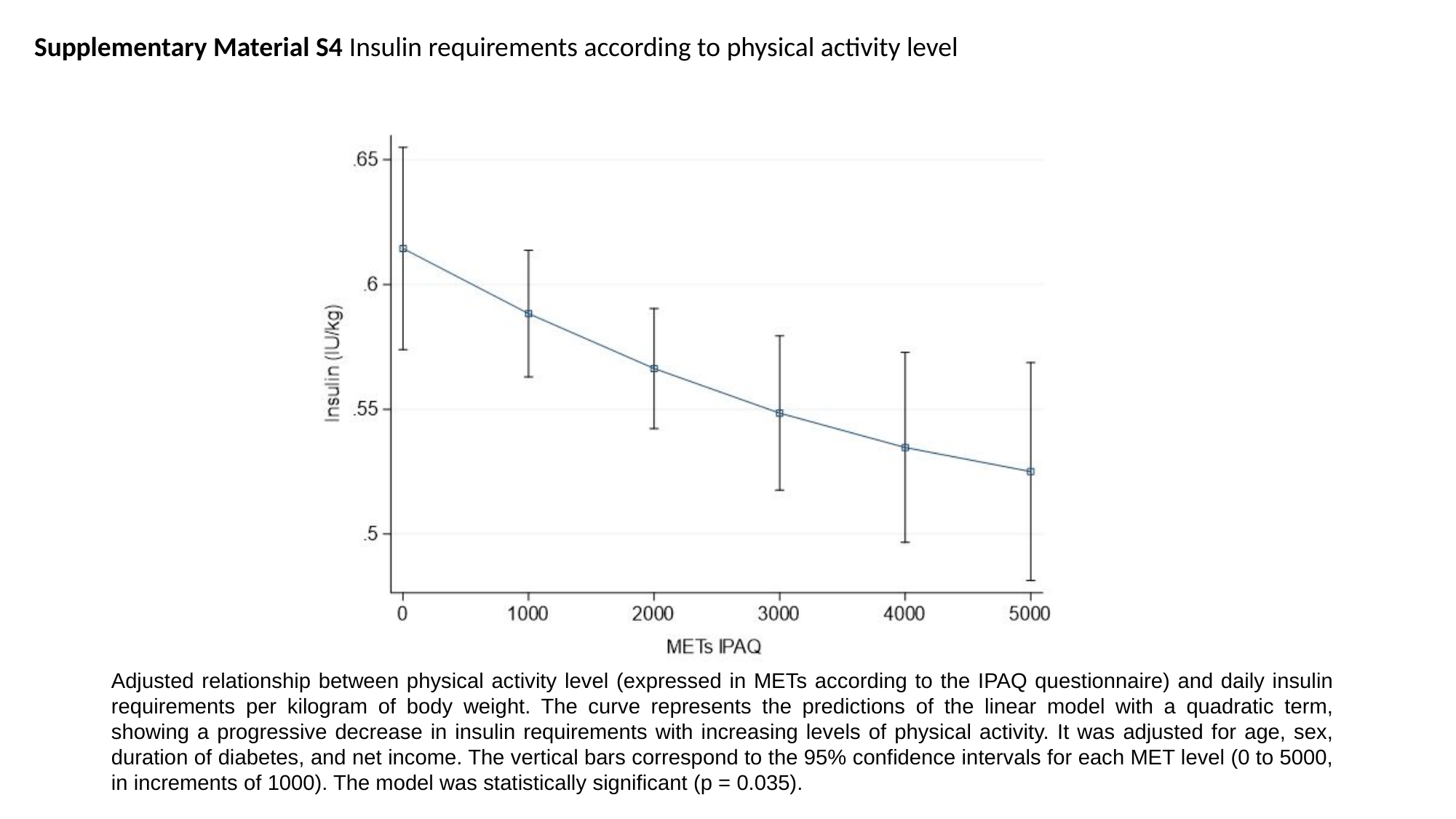

Supplementary Material S4 Insulin requirements according to physical activity level
Adjusted relationship between physical activity level (expressed in METs according to the IPAQ questionnaire) and daily insulin requirements per kilogram of body weight. The curve represents the predictions of the linear model with a quadratic term, showing a progressive decrease in insulin requirements with increasing levels of physical activity. It was adjusted for age, sex, duration of diabetes, and net income. The vertical bars correspond to the 95% confidence intervals for each MET level (0 to 5000, in increments of 1000). The model was statistically significant (p = 0.035).
