# Supplementary material for: Association of Physical Activity and Socioeconomic Status With Glycaemic Control in Adults With Type 1 Diabetes: A Cross‐Sectional Study Using CGM Data
Source: Diabetes Metab Res Rev. 2026 Feb 27;42(3):e70146. doi: 10.1002/dmrr.70146 (PMC12949369; doi:10.1002/dmrr.70146)
Supplement: Supplementary file 6 — Figure S5: Multivariable linear regression of insulin dose. [file DMRR-42-e70146-s008.pptx]

## Slide 1
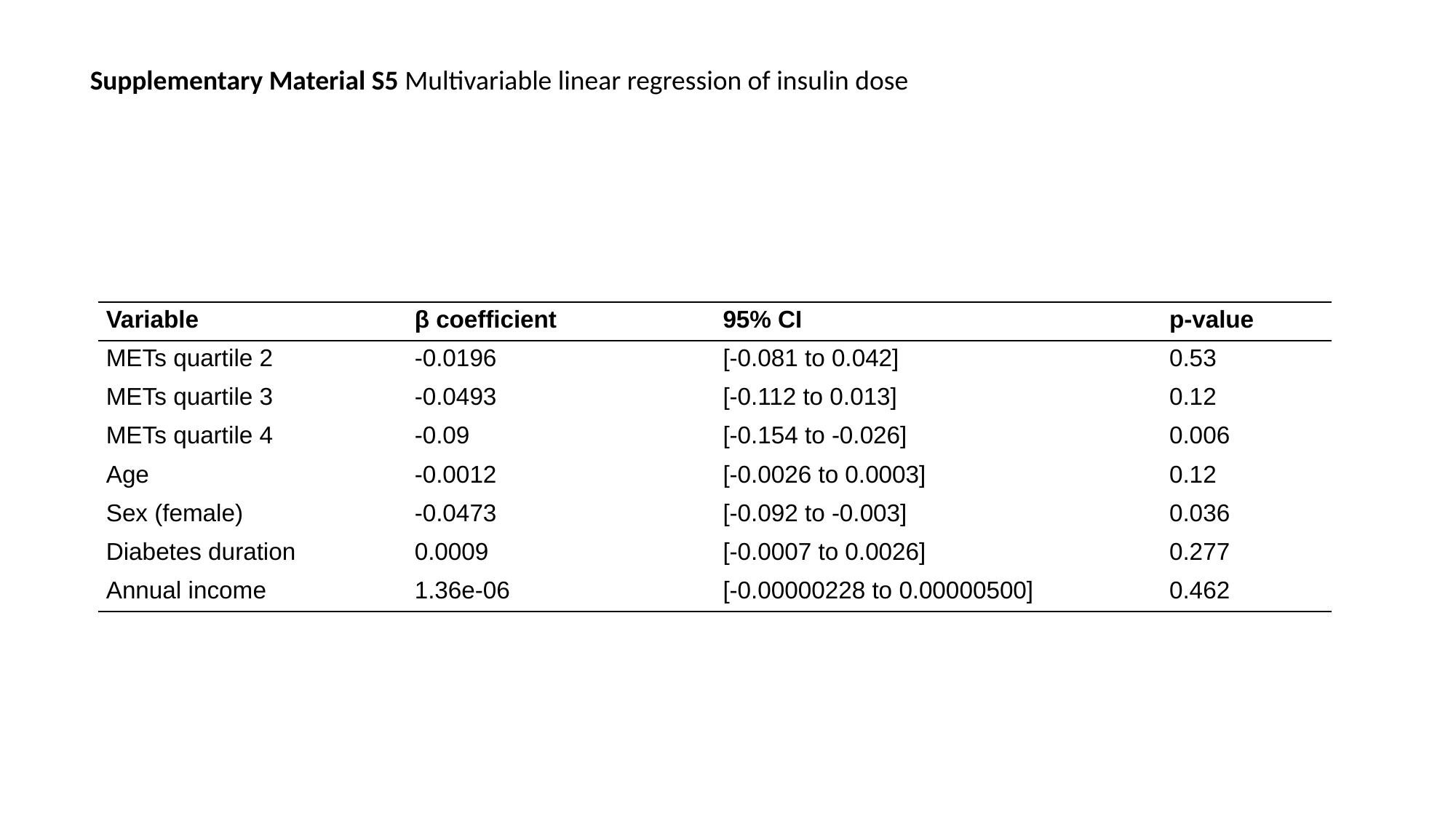

Supplementary Material S5 Multivariable linear regression of insulin dose
| Variable | β coefficient | 95% CI | p-value |
| --- | --- | --- | --- |
| METs quartile 2 | -0.0196 | [-0.081 to 0.042] | 0.53 |
| METs quartile 3 | -0.0493 | [-0.112 to 0.013] | 0.12 |
| METs quartile 4 | -0.09 | [-0.154 to -0.026] | 0.006 |
| Age | -0.0012 | [-0.0026 to 0.0003] | 0.12 |
| Sex (female) | -0.0473 | [-0.092 to -0.003] | 0.036 |
| Diabetes duration | 0.0009 | [-0.0007 to 0.0026] | 0.277 |
| Annual income | 1.36e-06 | [-0.00000228 to 0.00000500] | 0.462 |
