# Supplementary material for: Association of Physical Activity and Socioeconomic Status With Glycaemic Control in Adults With Type 1 Diabetes: A Cross‐Sectional Study Using CGM Data
Source: Diabetes Metab Res Rev. 2026 Feb 27;42(3):e70146. doi: 10.1002/dmrr.70146 (PMC12949369; doi:10.1002/dmrr.70146)
Supplement: Supplementary file 7 — Figure S6: Association between physical activity and lipid parameters. [file DMRR-42-e70146-s001.pptx]

## Slide 1
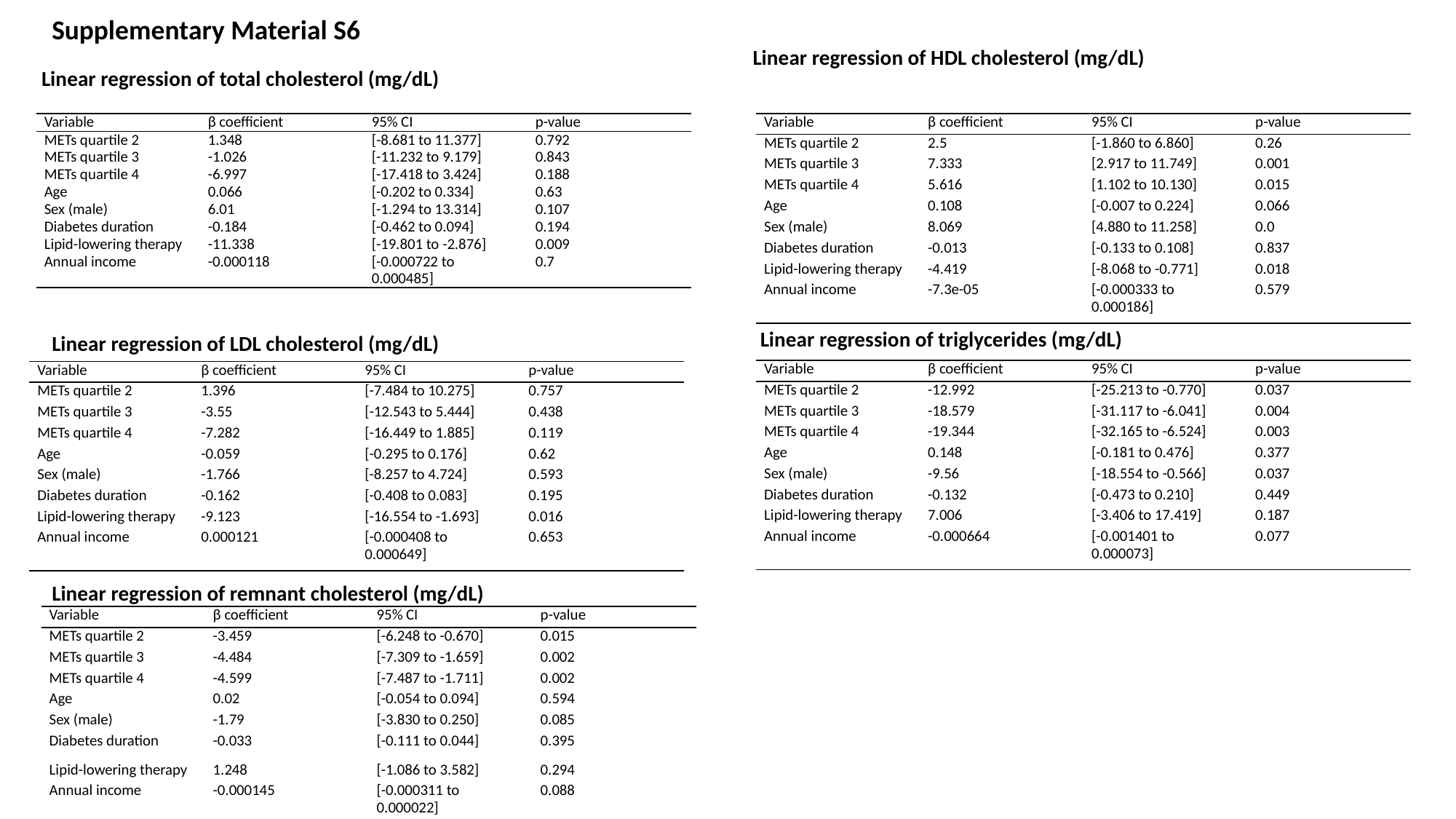

Supplementary Material S6
Linear regression of HDL cholesterol (mg/dL)
Linear regression of total cholesterol (mg/dL)
| Variable | β coefficient | 95% CI | p-value |
| --- | --- | --- | --- |
| METs quartile 2 | 1.348 | [-8.681 to 11.377] | 0.792 |
| METs quartile 3 | -1.026 | [-11.232 to 9.179] | 0.843 |
| METs quartile 4 | -6.997 | [-17.418 to 3.424] | 0.188 |
| Age | 0.066 | [-0.202 to 0.334] | 0.63 |
| Sex (male) | 6.01 | [-1.294 to 13.314] | 0.107 |
| Diabetes duration | -0.184 | [-0.462 to 0.094] | 0.194 |
| Lipid-lowering therapy | -11.338 | [-19.801 to -2.876] | 0.009 |
| Annual income | -0.000118 | [-0.000722 to 0.000485] | 0.7 |
| Variable | β coefficient | 95% CI | p-value |
| --- | --- | --- | --- |
| METs quartile 2 | 2.5 | [-1.860 to 6.860] | 0.26 |
| METs quartile 3 | 7.333 | [2.917 to 11.749] | 0.001 |
| METs quartile 4 | 5.616 | [1.102 to 10.130] | 0.015 |
| Age | 0.108 | [-0.007 to 0.224] | 0.066 |
| Sex (male) | 8.069 | [4.880 to 11.258] | 0.0 |
| Diabetes duration | -0.013 | [-0.133 to 0.108] | 0.837 |
| Lipid-lowering therapy | -4.419 | [-8.068 to -0.771] | 0.018 |
| Annual income | -7.3e-05 | [-0.000333 to 0.000186] | 0.579 |
 Linear regression of triglycerides (mg/dL)
Linear regression of LDL cholesterol (mg/dL)
| Variable | β coefficient | 95% CI | p-value |
| --- | --- | --- | --- |
| METs quartile 2 | -12.992 | [-25.213 to -0.770] | 0.037 |
| METs quartile 3 | -18.579 | [-31.117 to -6.041] | 0.004 |
| METs quartile 4 | -19.344 | [-32.165 to -6.524] | 0.003 |
| Age | 0.148 | [-0.181 to 0.476] | 0.377 |
| Sex (male) | -9.56 | [-18.554 to -0.566] | 0.037 |
| Diabetes duration | -0.132 | [-0.473 to 0.210] | 0.449 |
| Lipid-lowering therapy | 7.006 | [-3.406 to 17.419] | 0.187 |
| Annual income | -0.000664 | [-0.001401 to 0.000073] | 0.077 |
| Variable | β coefficient | 95% CI | p-value |
| --- | --- | --- | --- |
| METs quartile 2 | 1.396 | [-7.484 to 10.275] | 0.757 |
| METs quartile 3 | -3.55 | [-12.543 to 5.444] | 0.438 |
| METs quartile 4 | -7.282 | [-16.449 to 1.885] | 0.119 |
| Age | -0.059 | [-0.295 to 0.176] | 0.62 |
| Sex (male) | -1.766 | [-8.257 to 4.724] | 0.593 |
| Diabetes duration | -0.162 | [-0.408 to 0.083] | 0.195 |
| Lipid-lowering therapy | -9.123 | [-16.554 to -1.693] | 0.016 |
| Annual income | 0.000121 | [-0.000408 to 0.000649] | 0.653 |
Linear regression of remnant cholesterol (mg/dL)
| Variable | β coefficient | 95% CI | p-value |
| --- | --- | --- | --- |
| METs quartile 2 | -3.459 | [-6.248 to -0.670] | 0.015 |
| METs quartile 3 | -4.484 | [-7.309 to -1.659] | 0.002 |
| METs quartile 4 | -4.599 | [-7.487 to -1.711] | 0.002 |
| Age | 0.02 | [-0.054 to 0.094] | 0.594 |
| Sex (male) | -1.79 | [-3.830 to 0.250] | 0.085 |
| Diabetes duration | -0.033 | [-0.111 to 0.044] | 0.395 |
| Lipid-lowering therapy | 1.248 | [-1.086 to 3.582] | 0.294 |
| Annual income | -0.000145 | [-0.000311 to 0.000022] | 0.088 |
