# Supplementary material for: Association of Physical Activity and Socioeconomic Status With Glycaemic Control in Adults With Type 1 Diabetes: A Cross‐Sectional Study Using CGM Data
Source: Diabetes Metab Res Rev. 2026 Feb 27;42(3):e70146. doi: 10.1002/dmrr.70146 (PMC12949369; doi:10.1002/dmrr.70146)
Supplement: Supplementary file 9 — Table S2: AGReMA: A Guideline for Reporting Mediation Analyses. [file DMRR-42-e70146-s004.docx]

**Supplementary Table S2**

**AGReMA: A Guideline for Reporting Mediation Analyses**

| **Section/Topic** | **Item Number** | **Item Description** | **Reported on page No** |
| --- | --- | --- | --- |
| **Introduction** | | | |
| Objectives | 1 | State the objectives of the study specific to the mechanisms of interest. The objectives should specify whether the study aims to test or estimate the mechanistic effects | 4 |
| **Methods** | | | |
| Effects of interest | 2 | Specify the effects of interest | 5-6 |
| Causal assumptions | 3 | Specify assumptions about the causal model | 6 |
| Measurement | 4 | Clearly describe the interventions or exposures, mediators, outcomes, confounders, and moderators that were used in the analyses. Specify how and when they were measured, the measurement properties, and whether blinded assessment was used | 5-6 |
| Statistical methods | 5 | Describe the statistical methods used to estimate the causal relationships of interest. This description should specify analytical strategies used to reduce confounding, model building procedures, justification for the inclusion or exclusion of possible interaction terms, modelling assumptions, and methods used to handle missing data. Provide a reference to the statistical software and package used | 6 |
| **Results** | | | |
| Participants | 6 | Describe baseline characteristics of participants included in mediation analyses. Report the total sample size and number of participants lost during follow-up or with missing data | 7 |
| Outcomes and estimates | 7 | Report point estimates and uncertainty estimates for the exposure-mediator and mediator-outcome relationships. If inference concerning the causal relationship of interest is considered feasible given the causal assumptions, report the point estimate and uncertainty estimate | 8 |
| **Discussion** | | | |
| Limitations | 8 | Discuss the limitations of the study including potential sources of bias | 11 |
| Interpretation | 9 | Interpret the estimated effects considering the study’s magnitude and uncertainty, plausibility of the causal assumptions, limitations, generalizability of the findings, and results from relevant studies | 10-11 |

*From:*  Lee H, Cashin AG, Lamb SE, Hopewell S, Vansteelandt S, VanderWeele TJ, et al. A Guideline for Reporting Mediation Analyses of Randomized Trials and Observational Studies. The AGReMA Statement. JAMA. 2021;326(11):1045–1056. doi:10.1001/jama.2021.14075

AGReMA-SF is designed for articles that report mediation analyses of randomized trials or observational studies as a secondary focus of a paper. AGReMA-SF should be used in conjunction with CONSORT or STROBE for complete reporting.

For more information, visit: [agrema-statement.org](https://agrema-statement.org/)
